# Supplementary material for: Trapping single atoms on a nanophotonic circuit with configurable tweezer lattices
Source: Nat Commun. 2019 Apr 9;10:1647. doi: 10.1038/s41467-019-09635-7 (PMC6456496; doi:10.1038/s41467-019-09635-7)
Supplement: Supplementary file 1 — Supplementary Information [file 41467_2019_9635_MOESM1_ESM.pdf]

Supplementary Information: Trapping Single Atoms on A Nanophotonic Circuit with Configurable Tweezer Lattices

May E. Kim[1], Tzu-Han Chang[1], Brian M. Fields[1], Cheng-An Chen[1], and Chen-Lung Hung[1,2,3,*]

[1]*Department of Physics and Astronomy, Purdue University, 525 Northwestern ., West Lafayette, IN 47907*
[2]*Purdue Quantum Center, Purdue University, West Lafayette, IN 47907*
[3]*Birck Nanotechnology Center, Purdue University, 1205 W State St., West Lafayette, IN 47907*
[*]*clhung@purdue.edu*

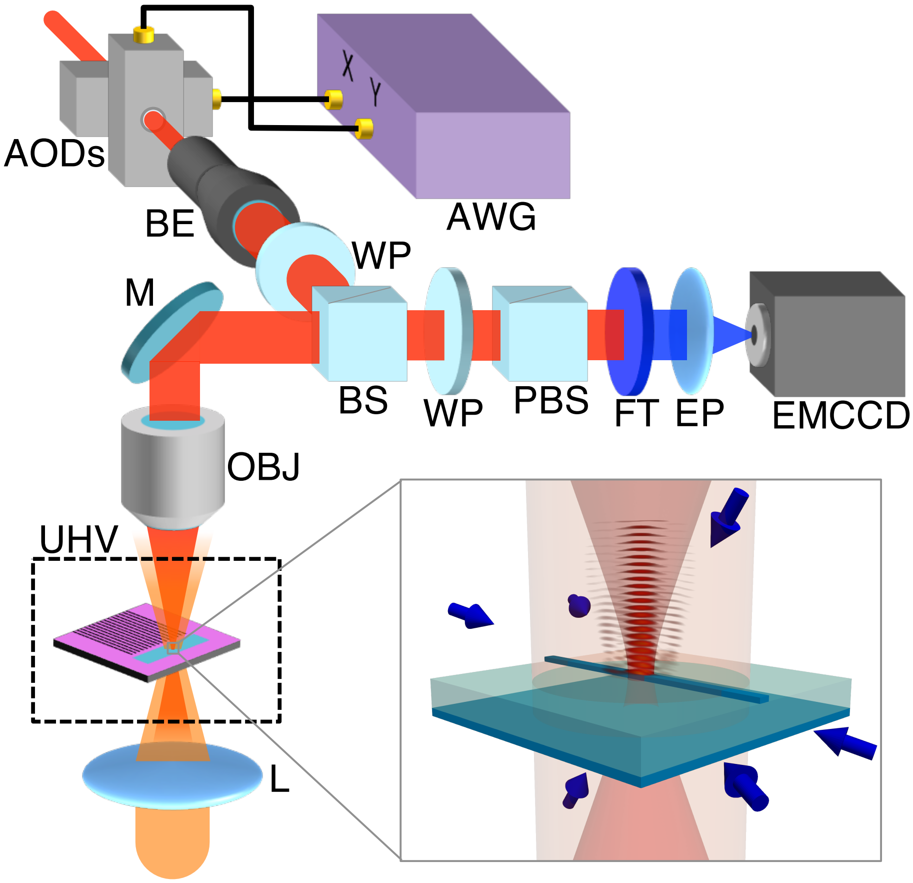

Supplementary Figure 1: **Schematic of the experiment apparatus.** Cold atoms are prepared on top of a photonic circuit in an ultrahigh vacuum chamber (dashed box marked by UHV) with three retro-reflected cooling beams (blue arrows) forming a magneto-optical trap. The tweezer beam is steered by two acousto-optic deflectors (AODs) that are driven by an arbitrary waveform generator (AWG), followed by beam expansion (BE) and projection through the microscope objective (OBJ). The bottom dipole beam is projected from the bottom of the membrane through a lens (L). Atomic fluorescence is filtered through waveplates (WP), a polarization beam splitter (PBS) and stacked interference filters (FT), and is recorded on an electron multiplying charge-coupled device (EMCCD) through an eyepiece (EP) that can independently adjust the image focal plane. Fluorescence imaging beams are shown in Fig. 1 and are not drawn here.

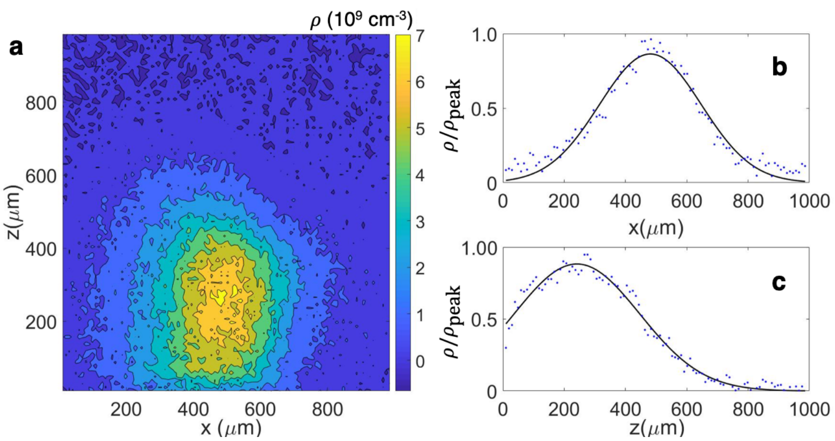

Supplementary Figure 2: **Atom number density near the membrane surface.** (a) On-chip atomic density distribution. Membrane surface is in the $z = 0$ plane. (b) and (c), Line-cuts of the density distribution through the cloud center, normalized by the peak density $\rho_{\rm peak} \approx 7 \times 10^9$ cm^{-3}. Projected atom number density at $z < 25$ μm near the chip surface is $\rho_0 \approx 3.5 \times 10^9$ cm^{-3}.

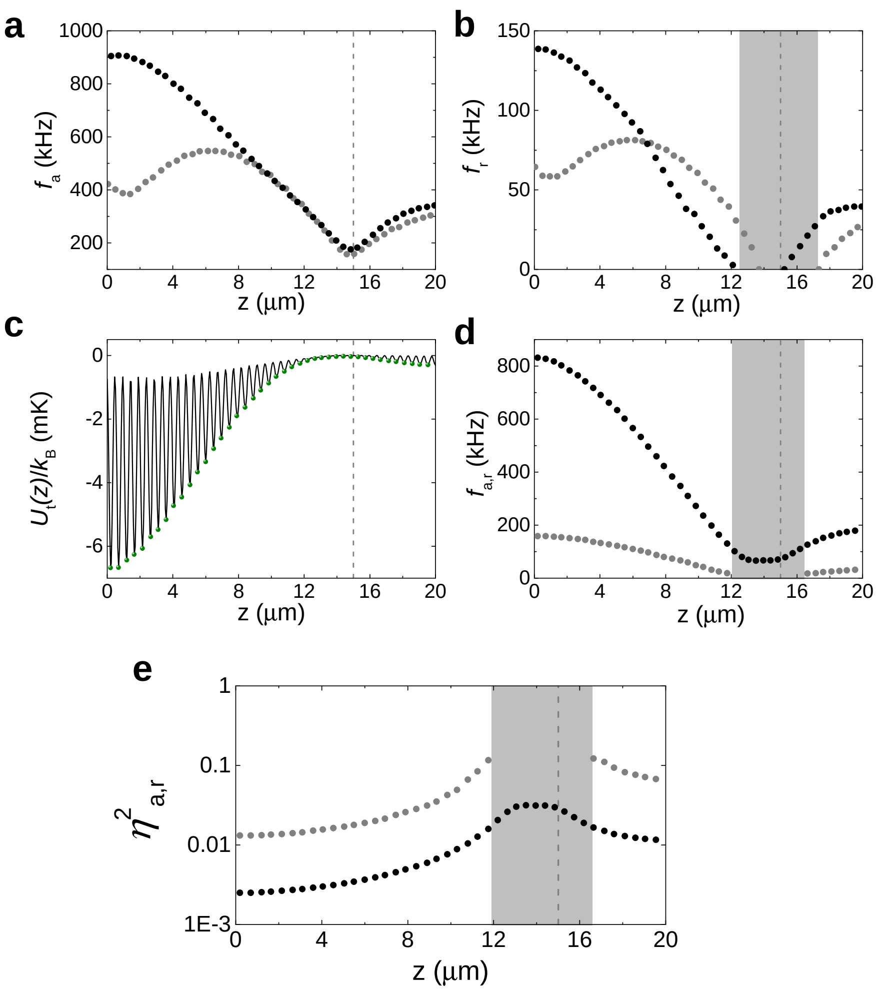

Supplementary Figure 3: **Trap frequencies and Lamb-Dicke parameters in a tweezer lattice on membrane.** (a-b) Axial and radial trap frequencies of the conveyor belt lattice potential, f_{a} and f_{r}, versus site position z when the bottom dipole beam is in-phase (black circles) and out-of-phase (gray circles), respectively. (c) Potential line-cut $U_{\mathrm{t}}(z)$ through the center of the stationary tweezer lattice formed by a 10 mW tweezer beam during fluorescence imaging, whose axial (black circles) and radial (gray circles) trap frequencies are plotted in (d). (e) The Lamb-Dicke parameters in the axial (black circles) and radial (gray circles) directions of the stationary tweezer lattice. The vertical dashed lines mark the position of the tweezer depth of field. The gray shaded areas mark the region with negative radial potential curvature and thus with no radial trapping.

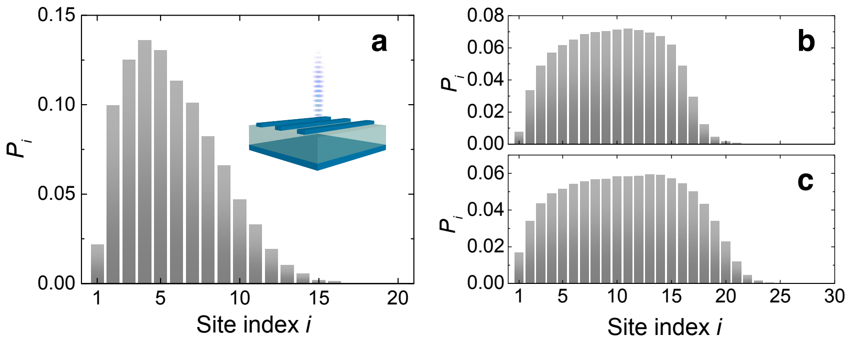

Supplementary Figure 4: **Monte Carlo simulation of trap loading probability in a tweezer lattice.** (a) Individual site loading probability on a waveguide as in Fig. 1. Inset depicts trap probability density above the waveguide. (b-c) Individual site loading probability in a conveyor belt on a membrane when the bottom dipole beam is in the in-phase (b) and the out-of-phase (c) conditions, respectively.
